# Targeted *In Vivo* Mutagenesis in Yeast Using CRISPR/Cas9 and Hyperactive Cytidine and Adenine Deaminases

**DOI:** 10.1021/acssynbio.2c00690

**Published:** 2023-07-24

**Authors:** Christos Skrekas, Angelo Limeta, Verena Siewers, Florian David

**Affiliations:** † Department of Life Sciences, Chalmers University of Technology, Gothenburg SE-41296, Sweden; ‡ Novo Nordisk Foundation Center for Biosustainability, Technical University of Denmark, DK-2800 Kgs. Lyngby, Denmark

## Abstract

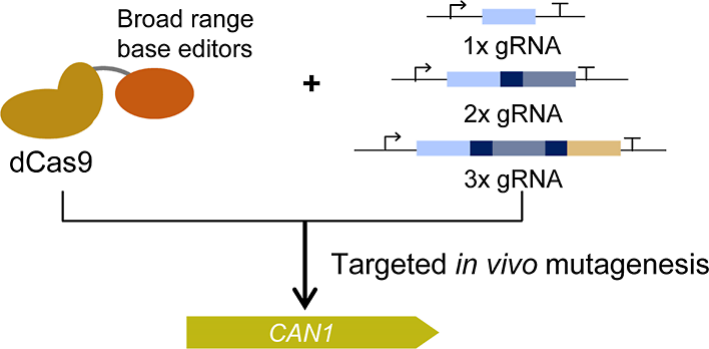

Directed evolution is a preferred strategy to improve the function of proteins such as enzymes that act as bottlenecks in metabolic pathways. Common directed evolution approaches rely on error-prone PCR-based libraries where the number of possible variants is usually limited by cellular transformation efficiencies. Targeted *in vivo* mutagenesis can advance directed evolution approaches and help to overcome limitations in library generation. In the current study, we aimed to develop a high-efficiency time-controllable targeted mutagenesis toolkit in the yeast *Saccharomyces cerevisiae* by employing the CRISPR/Cas9 technology. To that end, we fused the dCas9 protein with hyperactive variants of adenine and cytidine deaminases aiming to create an inducible CRISPR-based mutagenesis tool targeting a specific DNA sequence *in vivo* with extended editing windows and high mutagenesis efficiency. We also investigated the effect of guide RNA multiplexing on the mutagenesis efficiency both phenotypically and on the DNA level.

## Introduction

Directed evolution is a method used for improving or changing the properties of a protein of interest, and it usually involves several rounds of mutagenesis followed by selection for the desired traits.^1^ It has been used for applications such as the discovery of improved antibodies^2,3^ and in metabolic engineering approaches, evolving enzymes toward improved properties including substrate specificity and enzyme stability.^4,5^

Directed evolution usually relies on methods like error-prone PCR where genetic diversity is created *in vitro* through integration of random mutations into the gene of interest followed by mutant library generation and screening.^6,7^ The number of variants that can be tested by this method is restricted by the maximal possible library size generated.

An alternative to error-prone PCR based methods, particularly in *Saccharomyces cerevisiae*, is evolution via oligonucleotide transformation,^8,9^ where oligos carrying random mutations of the gene of interest are introduced into the yeast cells followed by enrichment of beneficial variants via selection assays. However, this approach does not allow for continuous evolution and the mutagenesis does not happen in parallel with cell growth. *In vivo* mutagenesis approaches are more suitable for continuous evolution since the generation of genetic diversity happens *in vivo* during cellular growth, which allows the mutagenesis and enrichment of beneficial mutations to happen in parallel or sequentially. Initially, *in vivo* mutagenesis approaches relied on random mutagenesis methods with the expression of mutagenic enzymes.^10,11^ However, since these methods target and cause defects to the DNA replication and repair mechanisms of the cell, mutations also occur outside the gene of interest and this could lead to lethal mutations or “parasite” mutations which can bypass the selection method and create false-positive variants.^12,13^ These issues can be addressed by targeting DNA-modifying enzymes directly to the gene of interest, thereby creating targeted directed *in vivo* mutagenesis systems.

A first approach of mutating a gene of choice *in vivo* has been developed based on an error-prone polymerase introducing mutations in the gene of interest during replication. This system, established in yeast, relies on an orthogonal replication system pairing the activity of an orthogonal error-prone DNA polymerase with the replication of a linear plasmid, harboring the gene of interest to be mutated.^14^ Although efficient, these approaches need elaborate pre-engineering and always target all genetic elements present on the plasmid, including promoters which might lead to increased numbers of false-positive variants. Also, these approaches can be used for evolving only the entire gene and not specific loci of a gene of interest, making such systems less suitable for evolution of certain parts of a protein of interest (e.g., the catalytic domain of an enzyme). Consequently, they are not ideal for evolving large multidomain genes in case only specific domains are aimed to be mutated. Therefore, there is a need for on-site-targeted *in vivo* mutagenesis systems which will be able to act directly on targeted genomic loci specified by the user.

CRISPR-based techniques offer a promising alternative for on-site targeted *in vivo* mutagenesis. These techniques exploit the precise targeting of a Cas9 chimeric protein with the use of a guide RNA of choice. The Cas9 variants that are used are either noncutting (dCas9) or single strand-cutting (nCas9). nCas9 can be fused with an error-prone DNA polymerase in order to initiate mutagenesis from the nick site.^15,16^ Also, dCas9 or nCas9 can be fused to DNA-modifying enzymes such as cytidine and adenine deaminases, and based on this approach, various site-directed *in vivo* mutagenesis tools have been developed both in mammalian cells^17,18^ and yeast.^19^

Activation-induced cytidine deaminase (AID) enzymes were originally identified to be participating in antibody somatic hypermutation, specifically deaminating cytosines (C) to uracils (U).^20−24^ AID is active on single-stranded DNA, and its activity is strongly connected to DNA transcription.^20,25^ Hess et al.^17^ successfully developed the targeted hypermutation tool CRISPR-X in mammalian cells. This tool uses a C-terminal truncated version of AID (AIDΔ) or its hyperactive variant AID*Δ. dCas9 is targeted to the DNA locus of interest by a guide RNA (gRNA) of choice. The AID domains are recruited via two MS2 hairpins, which are included in the respective gRNA.^26^ When the AID*Δ variant was used, an editing window of ±50 bp from the PAM site was achieved at a mutation rate of 1/500–1/1000 per bp (mutation rate during replication is around 1/10^9^ per bp). Nishida et al.^19^ have developed a similar tool in yeast by fusing the AID ortholog PmCDA1 to dCas9 using an extended 100 aa linker. The tool was found to mostly create targeted point mutations within a limited window (−13 bp to −20 bp from the PAM site). It was shown to be well suited for single-base editing but not for generation of mutations in broader editing windows, which would be needed for generation of genetic diversity and *in vivo* evolution applications.

Cytidine deaminases, even with increased efficiency and activity, bring a limitation to these mutagenesis systems because they mainly mutate cytidines. An addition to these systems that could lower this bias could be adenine deaminases, which mutate adenine (A) to guanine (G). TadA is originally an *Escherichia coli* tRNA adenine deaminase^27^ which was later evolved into the DNA-editing enzyme TadA*.^28^ TadA* has been further evolved^29^ for improved activity and Cas9 compatibility, resulting in the more active variant TadA8e, which in addition also shows limited off-target activity when coupled with dCas9. CRISPR base editing tools based on TadA8e have been used for precise genome editing in mammals^30^ and plants.^31−33^

In the present study, we aimed to develop CRISPR-based tools for the yeast *S. cerevisiae* that will be helpful for targeted *in vivo* mutagenesis with applications in controlled directed evolution approaches. The idea was to create base editors that mutate a defined genomic locus of interest guided by a gRNA of choice under controlled and inducible conditions. We first aimed to test the mutagenesis efficiency and the editing window of dCas9 fusions with the hyperactive cytidine deaminase AID*Δ and the hyperactive adenine deaminase TadA8e and its low off-target activity variant TadA8eV106W. We also sought to investigate how these base editors act when multiple gRNAs are expressed. To that end, we used endoribonuclease Csy4-mediated gRNA multiplexing, which has been proven efficient for gene editing and gene expression regulation approaches.^34,35^ Efficient gRNA multiplexing would make it possible to target different areas of the gene of interest simultaneously.

## Results

### dCas9-AID*Δ Creates Targeted Mutations in Broad Editing Windows in Yeast, and Its Effect Is Amplified by gRNA Multiplexing

**Figure 1:**
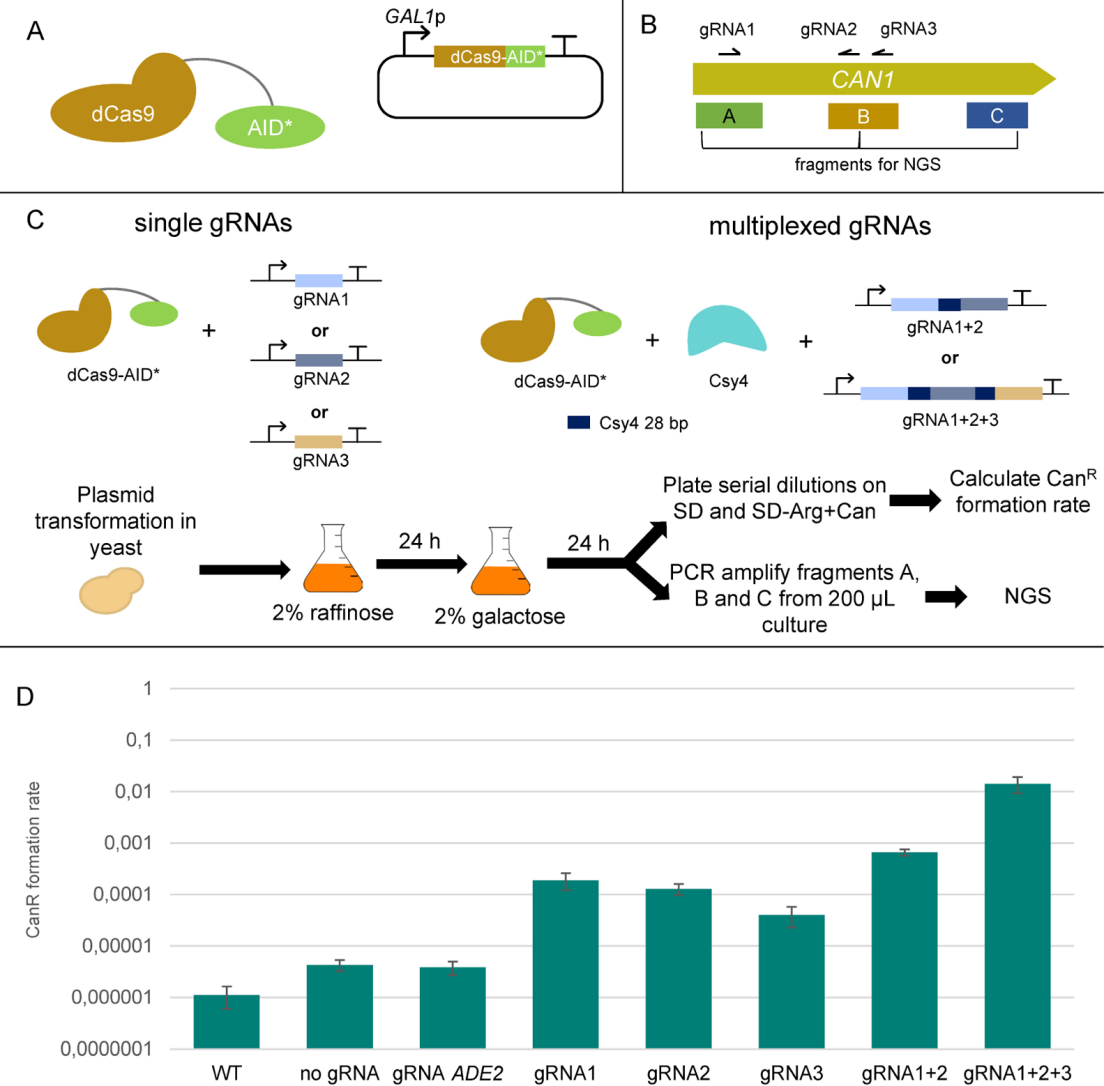
Overview of the dCas9-AID*Δ targeted mutagenesis assay. (A) Base editor design. dCas9 was fused to a flexible 100 aa linker and the hyperactive AID*Δ. The chimeric protein was expressed under the control of the *GAL1* promoter which offers inducible expression under galactose. (B) Overview of the *CAN1* gene and the three gRNAs designed to target it. A, B, and C fragments are 200 bp long and were selected for NGS for deep analysis of the mutagenesis. Fragment C was selected to estimate potential off-target mutagenesis. (C) Overview of the mutagenesis assay. dCas9-AID*Δ was expressed along with either single gRNAs or along with Csy4 ribonuclease and the gRNAs 1 and 2 multiplexed or all three gRNAs multiplexed. All the plasmids were introduced into yeast, precultures were grown in raffinose, and the main culture was grown in galactose for 24 h to induce mutagenesis. The phenotypical assay was done by serial dilution on plates with and without canavanine, and the ratio of Can^R^ cfu/total cfu was calculated to estimate the mutagenesis efficiency. Fragments A, B, and C were amplified and sent for NGS. (D) Can^R^ formation rate for all the conditions tested. For off-target activity estimation, a gRNA targeting the gene *ADE2* was used.

In the first part of the study, we aimed to investigate how CRISPR-mediated AID*Δ recruitment and induced mutagenesis work in yeast cells. To that end, we created a chimeric dCas9 fused with a codon-optimized AID*Δ domain connected through a flexible 100 aa linker.^19^ The expression was controlled via a galactose-inducible *GAL1* promoter. This enabled the specific temporal control of the mutagenesis step and decoupling it (Figure 1A) from the selection step, allowing targeted mutagenesis and enrichment for stable genotypes.

**Figure 2:**
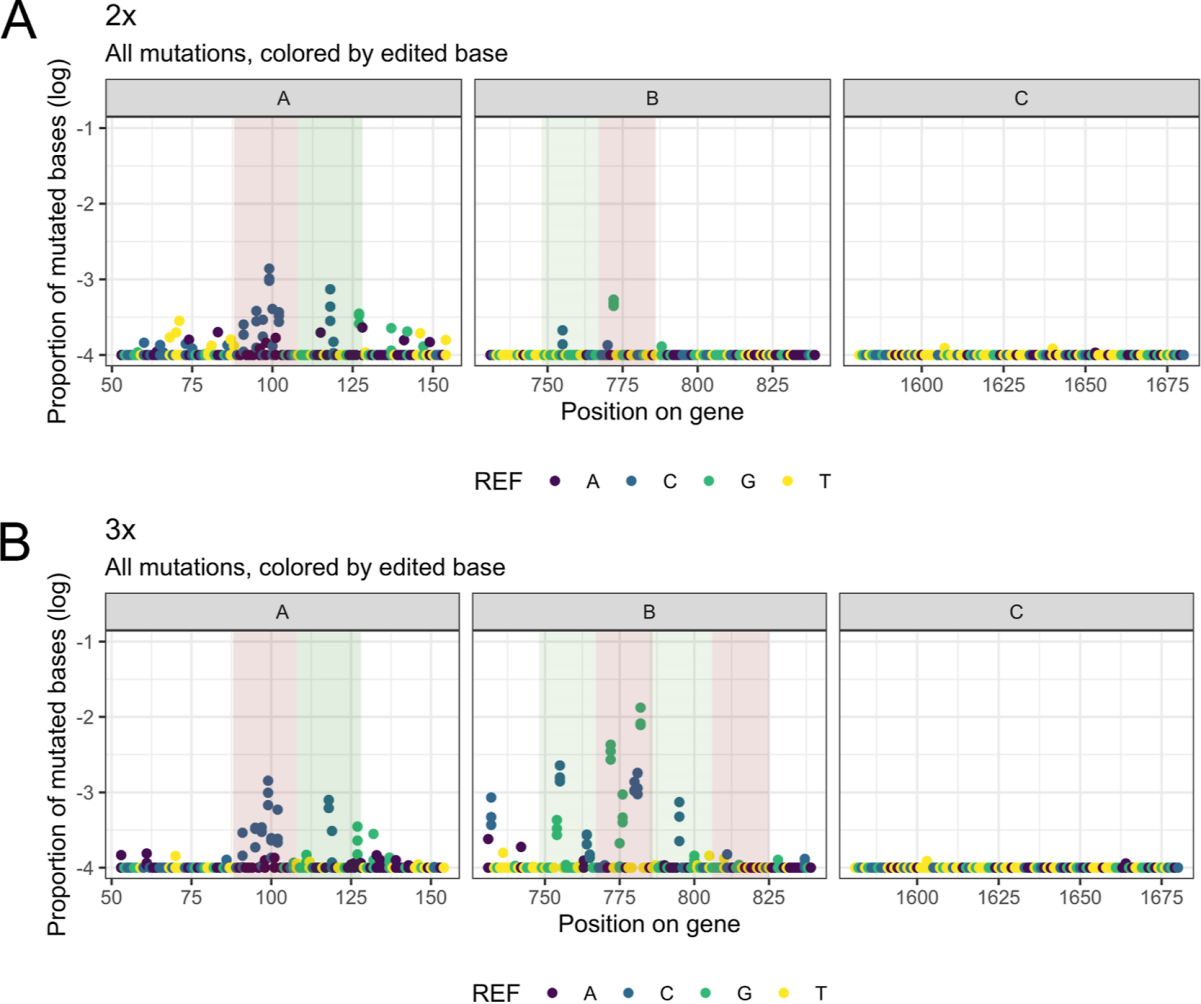
NGS results on the three fragments of the *CAN1* gene after mutagenesis with different combinations of gRNAs along with dCas9-AID*Δ. NGS fragments and gRNAs are shown in Figure 1B. The *x* axis of each graph denotes the gene position, and the *y* axis denotes the proportion of each mutation over the WT control in a logarithmic scale. The −20 bp region from the PAM site of each gRNA is shown in red, and the +20 bp region from the PAM site is shown in green. Each reference base that was mutated is shown with a different color. Mutation spectra of strains expressing the gRNAs 1 and 2 multiplexed (A) and all three gRNAs multiplexed (B) are shown.

We chose to test the mutagenesis efficiency phenotypically using the *CAN1* gene, coding for a plasma membrane arginine permease. A nonfunctional *can1* allele confers resistance to canavanine, which is easy to screen for.^19,36^ We designed three gRNAs directed toward *CAN1.* gRNA1 targets at the beginning of the gene (position 88–108 bp) and gRNA2 and gRNA3 target at the middle (positions 787–767 bp and 826–806 bp, respectively). We also defined three fragments of a size around 200 bp suitable for next-generation sequencing (NGS) to estimate the mutagenesis efficiency around each gRNA region (Figure 1A). Fragment A covers the positions 52–155 bp (gRNA1), fragment B covers the positions 730–840 bp (gRNA2 and gRNA3), and fragment C covers the positions 1580–1681 where no gRNA binds. Fragment C was selected for estimating the off-target effect of our base editor. The three gRNAs were expressed as single constructs but also multiplexed employing Csy4. Multiplexing combinations were gRNA1 along with gRNA2 and all three gRNAs combined (Figure 2C). In addition, we designed a gRNA targeting the *ADE2* gene to investigate potential off-target effects.

We screened the mutagenesis efficiency of dCas9-AID*Δ both phenotypically and genotypically. The expression of the base editor was induced by using galactose as a carbon source as outlined in the Materials and Methods section. The prevalence of Can^R^ colonies (cfu) over the total number of colonies appearing on SD media was used as a phenotypical indicator of mutagenesis activity. This number is also expressed as Can^R^ formation frequency. The results are shown in Figure 1D. The background Can^R^ formation frequency in the strain expressing no gRNA and no base editor was around 10^–6^ Can^R^/total cfu. This number was slightly increased when no gRNA or a gRNA that targets the *ADE2* gene was expressed together with the base editor. In case one of the three on-target gRNAs was expressed alone, the Can^R^ formation rates were similar for all tested gRNAs, around 10^–4^ Can^R^/total cfu. When the two first gRNAs were multiplexed, the Can^R^ formation rate was increased to 10^–3^ Can^R^/total cfu, and when all three gRNAs were multiplexed, it was further increased to 10^–2^ Can^R^/total cfu. These data indicate that gRNA multiplexing significantly elevates the mutagenesis efficiency of dCas9-AID*Δ.

The next step was to explore the targeted mutagenesis pattern and efficiency on a DNA level without any bias in relation to preselection of specific phenotypes. To that end, we analyzed the selected 200-bp fragments via deep sequencing. The analysis was directly performed on DNA derived from galactose-grown cells without any selection on canavanine in order to avoid bias toward functional mutations creating a Can^R^ phenotype. Canavanine resistance assays have been previously used for estimating the mutation rates in yeast.^37,38^ DNA sequencing reads were aligned to the *CAN1* locus, and the proportion of mutated bases was calculated for each position on the *CAN1* sequence (Figure 2, Methods). The results are shown in Figure 2. When a single gRNA was expressed, the mutagenesis efficiencies were low (Supporting Information Figure 1). In case two distant gRNAs (gRNA1 and gRNA2) were expressed simultaneously, the mutagenesis effect was increased in both loci in a region ±20 bp from the PAM site of each gRNA (Figure 2A). When all three gRNAs were multiplexed, the mutagenesis efficiency was further elevated, especially with regard to gRNAs 2 and 3 that are in close proximity (Figure 2B). These gRNAs bind in the same direction and their PAM sites have a distance of 39 bp from each other. The mutation frequency was highly increased, especially in the region between the two PAM sites.

**Figure 3:**
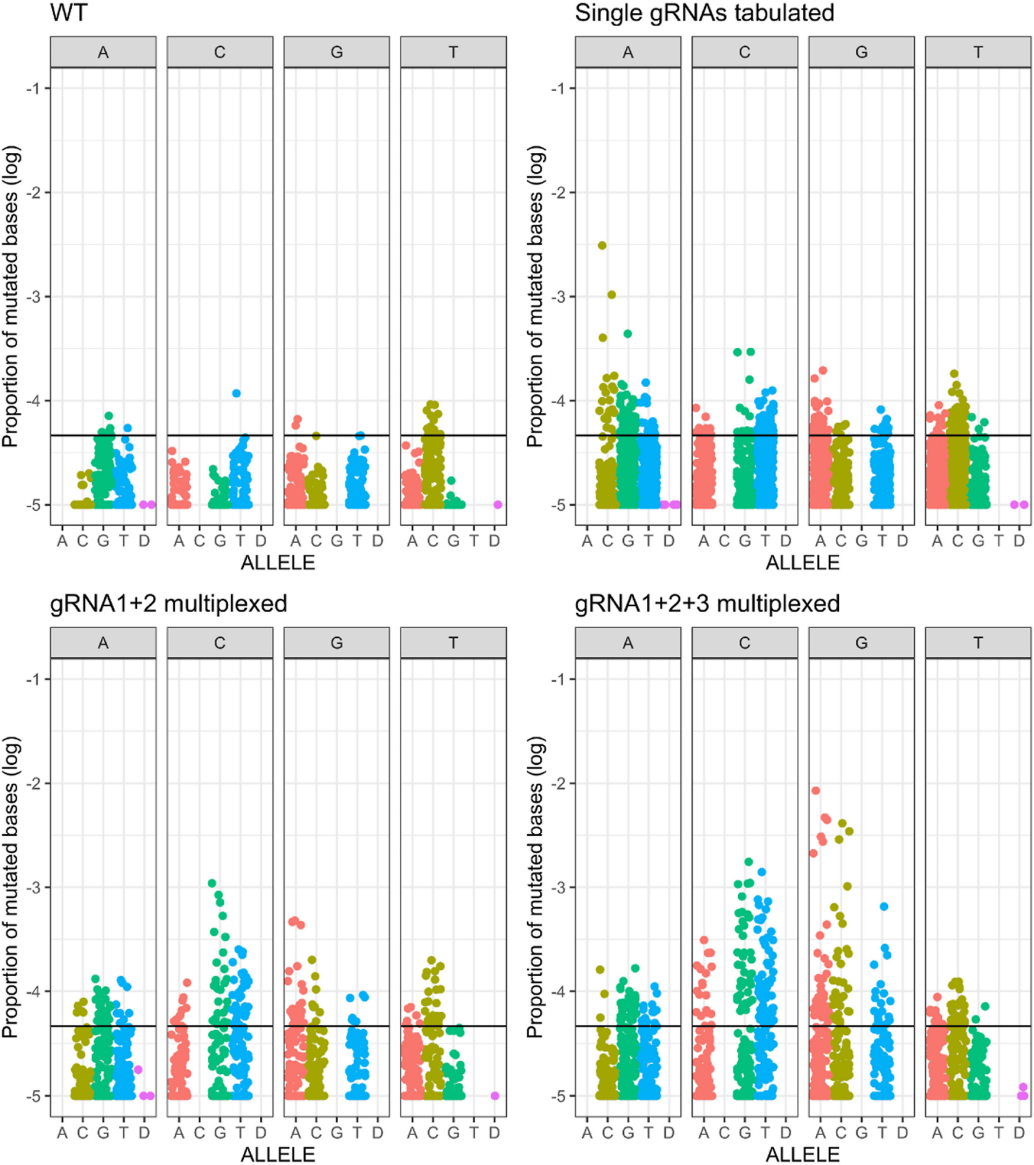
Base substitution frequencies of the different targeted mutagenesis experiments. The top left graph shows data from all WT samples. The black line indicates the region in which 95% of the events are located in the WT samples. Plotted is the proportion of mutated bases for each position in the *CAN1* target sequence grouped by the base substitution type, with each facet representing the reference sequence base and each color representing the resulting mutated base. The top right graph shows the base substitution frequencies for pooled data obtained from three single gRNA experiments in combination with dCas9-AID*Δ. The two bottom graphs show the base substitution frequencies caused by the expression of dCas9-AID*Δ and 2× multiplexed gRNAs (bottom left) and 3× multiplexed gRNAs (bottom right). D: deletion.

Additionally, we grouped the different mutations by nucleotide exchange for the different gRNA expression schemes (Figure 3). In the case of single gRNAs expressed, we observed a slight increase in all kinds of substitutions. When two gRNAs were multiplexed, we observed a further increase mostly with regard to C to G/T and G to A/C substitutions. When all three gRNAs were multiplexed, the C and G mutagenesis rates were elevated even further. It has to be noted that the highest increase in the mutagenesis rate was observed in the case of G to all other three bases. This could be explained by the fact that the two gRNAs with close proximity to each other bind both to the noncoding strand, so they are more likely to mutate C bases which appear as G mutations on the coding strand. This, along with the alignment results presented above, could be an additional indication that binding of two dCas9-AID*Δ in close proximity could have a synergistic effect and boost mutagenesis efficiency.

**Figure 4:**
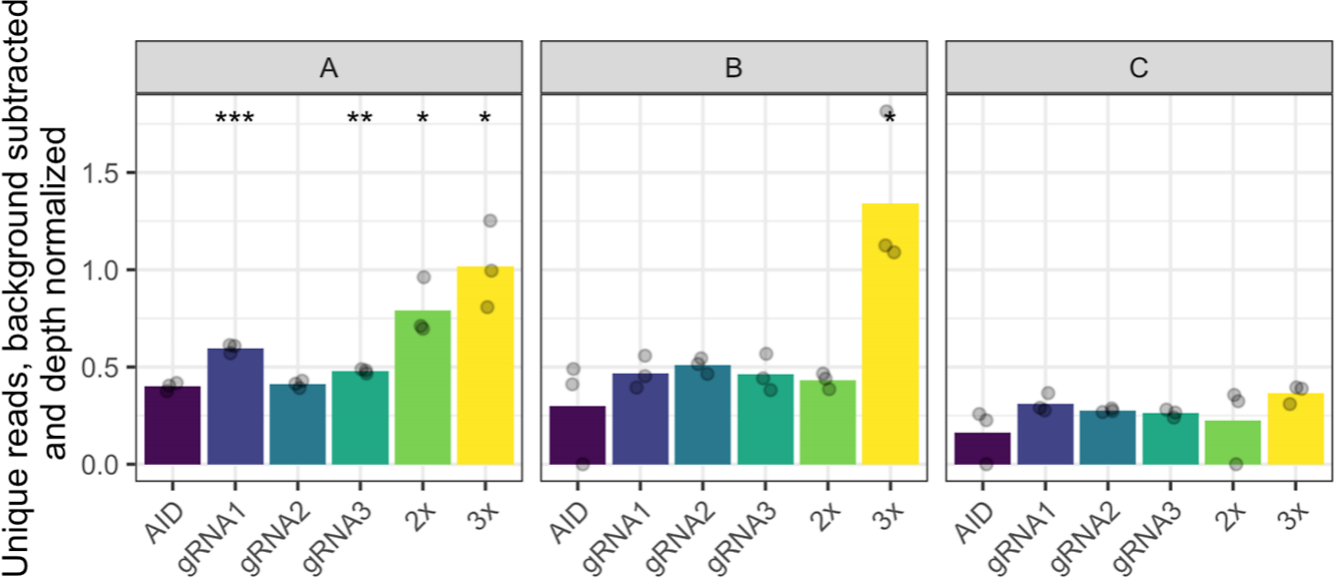
Number of unique NGS reads having a mutation in the dCas9-AID*Δ experimental dataset. Three fragments of the *CAN1* gene were used for NGS as defined in Figure 1B. Biological triplicates were analyzed for all samples. As unique reads, we define the reads that are different from the reads that occurred in the WT strain without the base editor and gRNA(s). The number of unique reads was normalized to the sequencing depth of each sample. The base editor was dCas9-AID*Δ, and the three gRNAs were expressed in three ways: individually, gRNA1+gRNA2 combined (2×), or gRNA1+gRNA2+gRNA3 combined (3×). The AID sample had only the base editor plasmid and no gRNA. The AID sample was used as a reference, and *p*-values were calculated for each sample. * *p*-value <0.05, ** *p*-value <0.01, and *** *p*-value <0.001.

For a closer insight into the mutagenesis patterns, we examined the unique mutations that were present in our NGS data on a read-by-read basis. We calculated the number of unique reads in each of our samples compared with the wild-type (WT) NGS data normalized by sequencing depth. We compared the number of unique reads that occurred in each dCas9-AID*Δ/gRNA combination with the unique reads in the strain that solely expressed the base editor plasmid (Figure 4). We can see that gRNA1 expression leads to some mutated bases on the gRNA binding site, but gRNAs 2 and 3 did not significantly increase the number of unique reads compared with when only the base editor was expressed. Combinatorial expression of gRNA1 (targeting NGS fragment A) and gRNA2 (targeting NGS fragment B) increased the number of unique reads even further only in fragment A. Combining the three gRNAs increased the number of unique reads in both fragments A and B. The increase was more pronounced in fragment B than in fragment A, something that could indicate that the proximity of the base editor binding sites can increase the base editing efficiency. However, the boost of unique reads that was observed in fragment A indicates that gRNA target proximity might not be the sole reason for the enhanced efficiency that was observed in the case of the triple gRNA expression. We further grouped our unique reads per number of single-nucleotide polymorphisms (SNPs) (Supporting Information Figure 3). The pattern of the results did not differ significantly from the previous analysis, and the majority of the unique reads had one or two SNPs. When all three gRNAs were expressed, we saw a small increase in unique reads with three SNPs which was significant (*p*-value <0.01) in fragment B where two proximal gRNAs were expressed.

Apart from deep sequencing, we also investigated the mutations present in clones that showed a Can^R^ phenotype. Only a nonfunctional *CAN1* gene confers canavanine resistance, so only mutants harboring at least one deleterious mutation were expected. We sequenced the *CAN1* open reading frame region of 20 Can^R^ colonies from each of the following strains: (a) empty plasmids, (b) base editor and gRNA1 single expressed, (c) base editor and gRNA1 and gRNA2 multiplexed, and (d) base editor with the three gRNAs multiplexed. Then, the mutations were identified and the mutation rate was calculated as a percentage of clones that carry the mutated base (Supporting Information Figure 2). In these data, we did not observe the intense boost in mutations when expressing two gRNAs in close proximity which we previously saw in deep sequencing. The highest mutation frequency was observed when a single gRNA (gRNA1) was expressed, and it was around 40%.

### dCas9-TadA8e and dCas9-TadA8e V106W Create Mutations with High Efficiency Using Multiplexed gRNA Expression

We sought to investigate whether the effect of multiplexed expression of gRNAs and the associated increase in mutagenesis efficiency is a general principle which could be also applied to other CRISPR base editors. To that end, we implemented another base editor in our system using the adenine deaminases TadA8e and TadA8eV106W. These adenine deaminases have shown broad editing windows in mammalian cells when coupled with dCas9, with the V106W variant showing decreased off-target activity.^29^ We created CRISPR base editors by fusing TadA8e and TadA8eV106 at the N-terminus of dCas9 linked by a 100 aa flexible linker, the same that was used in the AID*Δ experiments.

**Figure 5:**
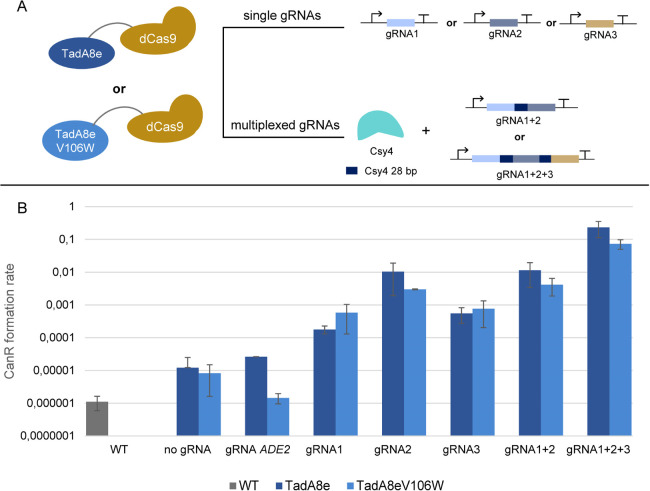
Overview of the adenine deaminase assay. (A) Hyperactive adenine deaminases TadA8e and TadA8e V106W were fused with a 100 aa flexible linker and dCas9. TadA8e V106W is reported to show decreased off-target activity. The same experimental strategy and the same gRNAs as in the AID*Δ assay (Figure 1) were used to estimate the mutagenesis efficiency. (B) Can^R^ formation rates for both TadA8e and TadA8e V106W and for all the experimental conditions tested.

First, a phenotypical screening was performed, following the same experimental design as conducted for dCas9-AID*Δ. We used the same gRNAs targeting the gene *CAN1*, expressed them as single constructs, dual multiplexed (gRNA1+2) and triplexed (gRNA1+2+3), and calculated the Can^R^ formation rates (Figure 5). Both base editors when expressed along with a single gRNA showed similar Can^R^ formation rates, which were a bit higher in the case of gRNA2. Simultaneous expression of gRNA1 and gRNA2 led to mutagenesis levels similar to gRNA2 alone, but when all three gRNAs were multiplexed, we saw a 10-fold increase of the Can^R^ formation rate compared to the most efficient gRNA for both base editors. In addition, a gRNA targeting the gene *ADE2* was used to estimate off-target efficiency. When *ADE2* targeting was used along with the TadA8eV106W-dCas9 base editor, the Can^R^ formation rate was at similar levels as the strain expressing no gRNA and base editor. On the contrary, the combination of the off-target gRNA and TadA8e-dCas9 variant led to about 10-fold more Can^R^ clones than the WT strain.

Subsequently, the mutation profile of Can^R^ mutants was investigated on the DNA level. To that end, a 500-bp sequence in the center of the *CAN1* gene (534–1011 bp) was selected for Sanger sequencing. In the middle of this fragment, gRNA2 and gRNA3 bind (Figure 5A). This region was chosen in order to investigate the base editing window and efficiency when gRNA2 only or both gRNA2 and gRNA3 are expressed.

**Figure 6:**
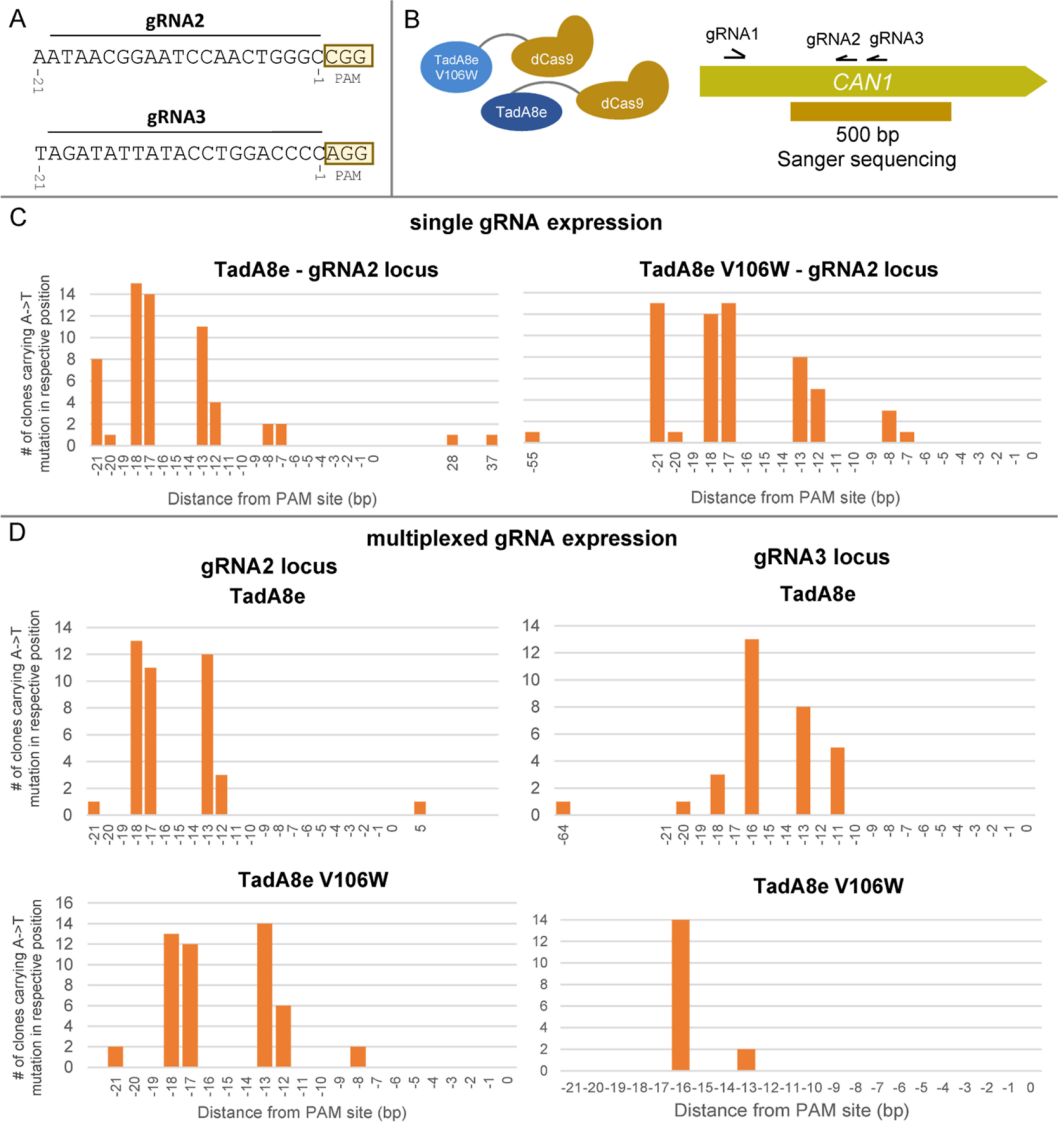
Overview of the mutations generated by TadA8e-dCas9 and TadA8eV106W-dCas9. (A) DNA sequences of the two gRNA binding loci that are examined in this experimental setup. In the yellow box, the PAM site of each locus is denoted. (B). For estimating mutagenesis efficiency for both TadA8e variants, a 500-bp part around the binding sites of gRNA2 and gRNA3 was amplified. The amplification was done on individual Can^R^ clones. For each condition, 15 clones were screened. (C) Mutation spectra for both variants when a single gRNA (gRNA2) was expressed. (D) Mutagenesis effect for gRNA2 and gRNA3 loci when gRNAs 1, 2, and 3 were multiplexed.

A total number of 15 Can^R^ clones was sequenced, sourcing from the strains expressing the adenine deaminase base editors and gRNA2 and 15 Can^R^ clones from the strains with the base editors and the triplicated gRNAs. Mutations were almost exclusively detected in the gRNA binding site, and the only types of mutations observed were T → C substitutions in the coding strand or A → G substitutions in the noncoding strand. Figure 6B shows the editing window of gRNA2 when TadA8e-dCas9 or TadA8eV106W-dCas9 was expressed. Editing starts at position −7 from the PAM site, and the highest mutation rate observed was at position −18. In the case of TadA8eV106W-dCas9, high mutation rates were observed also at position −21. Moreover, in the case of TadA8e-dCas9, two single mutations at a larger distance from the PAM site were observed at the positions +28 and +37 from the PAM site.

When the two proximal gRNAs 2 and 3 were expressed simultaneously, the gRNA2-binding nucleotides that are the closest to the PAM site were no longer mutated, but the mutagenesis rate of the positions −12/–13 and −17/–18 remained almost the same. gRNA3 led to a similar mutagenesis profile, especially when expressed together with TadA8e-dCas9. In the case of TadA8eV106W-dCas9, only two positions in the gRNA3 binding site were mutated, mostly position −16. The V106W mutation significantly lowered the off-target activity of TadA8e-dCas9 but also seemed to lower the on-target mutagenesis efficiency, especially in the context of multiplexed gRNA expression. gRNA3 had a slightly lower on-target score than gRNA2 (59.3 vs 62.4, respectively), but it is unclear if this difference can fully explain the low mutagenesis rate observed in the gRNA3 position when multiplexed gRNAs were expressed along with TadA8eV106W-dCas9.

## Discussion

In this study, we examined the performance of high-efficiency variants of cytidine deaminase (AID*Δ) and adenine deaminase (TadA8e and TadA8eV106W) for targeted *in vivo* mutagenesis in yeast cells. We sought to develop targeted mutagenesis systems based on CRISPR-based recruitment of these base editing enzymes. To that end, we fused these variants to dCas9 and estimated their mutagenesis efficiency on a target gene both phenotypically and on the DNA level. We also investigated the effect of multiplexed gRNA expression targeting a gene of interest to see whether mutation efficiencies could be increased.

Regarding the hyperactive variant AID*Δ, we saw a deaminase activity in an editing window narrower than what was observed in mammalian cells ( ±50bp)^17^ and approximately ±20 bp from the PAM site, which was increased by gRNA multiplexing. The base editing efficiency was significantly increased when two proximal gRNAs and a distant one (three gRNAs in total) were expressed simultaneously. The number of unique reads carrying an SNP was further increased when multiple gRNAs were expressed, and the increase became even more pronounced when two proximal gRNAs were expressed, also resulting in a higher number of SNPs. C and G mutagenesis to all other three bases also followed a similar pattern, and it was most evident when the three gRNAs were expressed simultaneously.

These findings could be connected to an effect based on AID*Δ dimerization. It has been shown via immunoprecipitation assays that AID can form dimeric or even multimeric complexes.^39^ Moreover, crystal structure analysis of another member of the cytidine deaminase family, APO2, revealed a dimerization domain that shares a high identity with a domain in AID. Point mutations on the most conserved residues showed nondetectable or significantly reduced deaminase activity.^40^ On the other hand, *in vitro* atomic force microscopy experiments have shown that AID along with single-stranded DNA is predominantly present as a monomer and shows deamination activity.^41^ These findings are in contradiction to whether AID operates in a monomer or dimer/multimer form to have functional deaminase activity. Hess et al.^17^ recruited AID*Δ in mammalian cells by adding two MS2-binding hairpins to the gRNA and fusing an MS2 domain to AID*Δ. In this case, the free AID*Δ protein could potentially form dimers or multimers before binding to the RNA hairpins, and this could be a reason for their high performance both in terms of increased mutation efficiency and larger editing window. Similar CRISPR RNA scaffolds have already been characterized in yeast for recruitment of transcription factors,^42^ and they could be also used for the recruitment of AID*Δ. Scaffold-mediated recruitment of AID*Δ in yeast cells could give a better insight into the real potential of this hyperactive mutant as a targeted *in vivo* gene diversification tool. In addition, we observed increased mutagenesis efficiency also when two remote gRNAs were expressed simultaneously. Lateral diffusion has been proposed as a potential mechanism that Cas9 uses in order to scan a target DNA sequence for PAM sites.^43^ The results of this previous study indicate that if a target sequence contains more than one PAM site at a close distance, Cas9 binds to the DNA for a longer time. Scanning for PAM sites in close proximity might increase the editing window and further contribute to the observed synergistic effects. However, we suggest that gRNA targeting sites in proximity and potential AID dimerization effects are the main contributors to the observed synergistic effects. Moreover, our Cas9-AID*Δ base editor shows a bias toward G/C mutations, and it still remains challenging to increase the mutagenesis efficiency on A/T nucleotides.

dCas9-AID* activity was analyzed by NGS of nonselected cells and Sanger sequencing of clones that showed a Can^R^ phenotype. These two analyses showed some contradicting results. For example, in the NGS data, poor mutagenesis rates on the targeted genomic sites were observed, but when Can^R^ clones were sequenced, we saw clear mutations in a region larger than ±20 bp from the PAM site of the gRNA. Additionally, in the case of multiple gRNA expression, Sanger-sequenced Can^R^ clones showed less mutation variability than the NGS reads, but in this case, the dataset was also much smaller. This can be explained by the fact that screening of clones that are selected based on a phenotype caused by certain types of mutations is biased and might filter out a large number of mutations that do not lead to the phenotype screened for. On the other hand, selection can be useful to identify mutations that occur in a lower frequency and may not be detected by NGS of nonselected cells.

Targeted base editors based on the hyperactive adenine deaminase variants TadA8e and TadA8eV106W showed a high mutation efficiency, which was restricted almost exclusively to the gRNA binding site. Moreover, gRNA multiplexing resulted in enhancement of the mutagenesis effect with both base editors, something that is reflected in the increase of the Can^R^ frequency that occurs upon multiple gRNA expression. Interestingly, the editing window appears to be expanded compared with the one previously observed in mammalian cells when a shorter (32 aa) linker was used to fuse the adenine deaminase with dCas9.^29^ In mammalian cells, the editing window was 11 bp, and in this study, it reached up to 14 bp. The V106W mutation seems to lower the off-target activity also in yeast, which makes this variant more suitable for targeted mutagenesis. These base editors, apart from the relatively narrow base editing window, showed a strong bias toward A → G mutations, which were the only ones observed in our sequencing results. However, since our study was based on a small dataset of colonies that showed a specific phenotype, a more extensive study with no bias and deep sequencing data could give a better insight into the activity and editing windows of those adenine deaminases in yeast.

In conclusion, this study gives a first insight into how hyperactive cytidine and adenine deaminases can be used for developing CRISPR-based targeted *in vivo* mutagenesis tools in yeast. Such base editors can be used for the targeted evolution of genes of interest, either in their full length or of selected domains. High-performance CRISPR-based base editors can be combined with small gRNA libraries—single or even multiplexed—that cover a gene of interest.^44,45^ The base editor expression can be induced, and a pool of specific variants can be created, which can be screened, e.g., for improved cellular growth or production.^46^ Our results show that mutagenesis efficiency varies depending on the gRNA used. Thus, when designing *in vivo* evolution assays, it is important to have a gRNA library that sufficiently covers the DNA region that should be mutagenized. It remains to be examined whether combinatorial recruitment of the cytidine and adenine deaminases can contribute to a less biased base editor which will mutate both C–G and A–T base pairs with similar chances. Similar approaches have been already successfully established in the form of protein fusions,^47^ but it would be interesting to implement this combinatorial strategy with stem loop-based approaches, allowing for the recruitment of multiple copies of base editors in a specific genomic locus.

## Materials and Methods

### Strains and Media

The background *S. cerevisiae* strain used in this study was CEN.PK113-11C (*MAT*a *MAL2-8C SUC2 ura3-52 his3Δ*).

All yeast strains were grown in a synthetic medium^48^ containing 20 g/L glucose, 7.5 g/L (NH_4_)_2_SO_4_, 14.4 g/L KH_2_PO_4_, 0.5 g/L MgSO_4_·7H_2_O, 1 mL/L vitamin mix, and 2 mL/L trace metal solution. The pH was adjusted to 6.5. The trace metal solution contained 15.0 g/L EDTA (disodium salt), 4.5 g/L ZnSO_4_·7H_2_O, 0.84 g/L MnCl_2_·2H_2_O, 0.3 g/L CoCl_2_·6H_2_O, 0.3 g/L CuSO_4_·5H_2_O, 0.4 g/L Na_2_MoO_4_·2H_2_O, 4.5 g/L CaCl_2_·2H_2_O, 3 g/L FeSO_4_·7H_2_O, 1g/L H_3_BO_3_, and 0.1 g/L KI. The vitamin solution contained 0.05 g/L biotin, 0.2 g/L 4-aminobenzoic acid, 1 g/L nicotinic acid, 1 g/L calcium pantothenate, 1 g/L pyridoxine-HCl, 1 g/L thiamine-HCl, and 25 g/L *myo*-inositol. When needed, histidine and/or uracil were added to the medium for auxotrophy supplementation at a concentration of 100 mg/L. The carbon source in some experiments was changed to 20 g/L galactose or 20 g/L raffinose when indicated. Liquid yeast cultures were grown in shake flasks at 30 °C with shaking at 200 rpm.

When plasmid selection was needed, yeast was grown on SD-His-Ura agar plates consisting of 6.9 g/L yeast nitrogen base without amino acids (Formedium), 0.77 g/L complete supplement mixture without histidine and uracil (Formedium), 20 g/L glucose, and 20 g/L agar. When no selection was needed, yeast was grown on SD agar plates which had the same composition as above apart from the complete supplement which had no dropouts and a concentration of 0.79 g/L. For selection of yeast clones that show a Can^R^ phenotype, SD-Arg+Can agar plates were used consisting of 6.9 g/L yeast nitrogen base without amino acids (Formedium), 0.74 g/L complete supplement mixture without arginine (Formedium), 20 g/L glucose, 60 mg/L canavanine, and 20 g/L agar.

For plasmid cloning and amplification, *E. coli* strain DH5α was used and grown in an LB medium consisting of 10 g/L sodium chloride, 5 g/L yeast extract, and 10 g/L peptone from casein. For agar plates, 16 g/L agar was added. For plasmid selection, antibiotics were added in the following concentrations: ampicillin 100 mg/L, kanamycin 50 mg/L, and chloramphenicol 25 mg/L. Liquid cultures were grown at 37 °C and 200 rpm, and agar plates were incubated at 37 °C for 16–20h.

### DNA Manipulation and Plasmid Construction

*E. coli* transformation was done by chemical transformation as previously described.^49^ For *S. cerevisiae* transformation, the LiAc/PEG chemical transformation method^50^ was followed. For PCR purifications, gel extractions and plasmid minipreps GeneJet kits were used (Thermo Fisher Scientific). Total DNA extraction from liquid *S. cerevisiae* cultures was done as described by Lõoke et al*.*^51^ PCR amplifications were performed with Phusion High-Fidelity DNA polymerase (Thermo Fisher Scientific) following the instructions of the manufacturer.

All the plasmids used in the current study were constructed with the use of the yeast modular cloning system MoClo following the vector design and the strategies that were previously described.^52,53^ Plasmid construction was done following one-pot Golden Gate assembly with T4 ligase (Thermo Fisher Scientific) and restriction enzymes Eco31I (BsaI) (Thermo Fisher Scientific) or Esp3I (BsmBI) (Thermo Fisher Scientific), and a previously described assembly protocol was followed.^53^ For the dCas9-AID*Δ plasmid construction, dCas9 was cloned in a part plasmid as a 3a part without the stop codon, 100 aa linker as a 3b part (amplified from pRS315e_pGal-dCas9-PmCDA1^19^), and AID*Δ with the stop codon as a 4a part. The parts were PCR-amplified and cloned into the MoClo part plasmid entry vector.^52^ These plasmid parts were combined in a one-pot Golden Gate assay to construct the plasmid LS-dCas9-AID*Δ-R1 with the *HIS3* marker and CEN/ARS origin of replication. For the adenine deaminase plasmid construction, the TadA8e or TadA8eV106W encoding gene was cloned as a 3a part without the stop codon and dCas9 was cloned as a 4a part with the stop codon. These parts were synthesized and cloned by Twist Biosciences (South San Francisco, CA). The plasmid parts were combined along with the 100-aa linker (3b part), resulting in the plasmids LS-TadA8e-dCas9-R1 and LS-TadA8eV106W-dCas9-R1. When Csy4-multiplexed gRNA arrays were used, the LS/R1 base editor plasmids were combined with the plasmid L1-Csy4-RE^53^ to construct a multicassette plasmid which had the base editor, a Csy4 ribonuclease expression cassette, *HIS3* marker, and CEN/ARS origin of replication.

gRNAs used in this study were designed using Benchling (www.benchling.com). Off-target and on-target scores for each gRNA were calculated based on the model of Doench et al*.*^54^ The gRNAs used in this study with their off-target and on-target scores are shown in Supporting Information Table S1. Single and Csy4-multiplexed gRNA cloning was done using pMCL9 as a cloning vector following the methodology described previously.^53^ The plasmids constructed in the current study are summarized in Supporting Information Table S3, and the primers used are summarized in Supporting Information Table S2.

### Canavanine Resistance Assays

Three different colonies of yeast strains CEN.PK113-11C containing a base editor plasmid and a gRNA plasmid (single or multiplexed) were cultivated for 24 h in synthetic media containing 2% raffinose. Then, the cultures were transferred to synthetic media containing 2% galactose in an initial OD of 0.1, and they were cultivated for 24 h. Subsequently, 100 μL of each culture was serially diluted until 10^–6^ and four 10 μL drops per dilution were plated on SD and SD-Arg+canavanine agar plates. This experimental design resulted in four technical replicates and three biological replicates per strain. After 3 days, the numbers of total cfu/mL and Can^R^ cfu/mL were calculated. The mutagenesis rate for each strain was calculated by dividing the Can^R^ cfu by the total cfu, and they were plotted in a logarithmic scale.

### NGS Sample Preparation and Data Analysis

200 μL from the 2% galactose cultures of the canavanine resistance assay described above was used for total DNA extraction. The three 200 bp fragments (A, B, and C) of the *CAN1* locus were PCR-amplified, and the primers can be found in Supporting Information Table S2. Then, the PCR fragments were cleaned up, indexed, and pooled based on Illumina DNA Nextera Sequencing as described by Lee et al*.*^55^ The pooled library was subjected to NGS using a MiSeq Benchtop Sequencer (Illumina, San Diego, CA).

The three sequenced regions on the *CAN1* locus were short enough to allow a complete overlap of complementary paired-end reads. Using NGmerge,^56^ paired-end reads in the FASTQ format with perfectly overlapping complementary regions were merged using the following parameters: mismatches to allow in the overlapping region = 0, FASTQ quality offset = 33, and maximum input quality score = 40. Merged reads were subsequently aligned to the *CAN1* locus using the burrows-wheeler aligner,^57^ and the resulting alignment files in BAM format were then sorted using SAMtools sort.^58^ Next, sorted BAM files were piled up into the tabular VCF format, containing counts of all detected genetic variants across samples, using BCFtools mpileup^58^ with the parameter maximum depth = 600 000. The entire pipeline for producing VCF files from raw FASTQ files was implemented using the Snakemake workflow engine,^59^ and package versions were managed using Conda.

Subsequent processing of the VCF file was performed in the R software package (version 4.1.2) along with the Tidyverse packages.^60^ The resulting VCF file was parsed into a tidy format containing allele counts for each position on the *CAN1* gene across all samples. Regions containing a read depth of less than 50 000 were discarded from the analysis. The ends of each of the three sequenced regions in the *CAN1* gene were also removed. Counts of nonreference bases at each position were then normalized for sequencing depth in order to calculate the proportion of mutated bases at each position across samples. Similarly, read-depth normalized proportions of each possible base substitution were also calculated. These values were then subtracted by the proportion of mutated bases in the WT sample in order to highlight regions enriched for mutations; negative values were set to zero.

In order to examine the number and diversity of generated mutations in individual yeast cells more closely, we examined read mutation distributions across all samples on a read-by-read basis. Starting from the aligned BAM files, as described above, sample-specific BAM files were loaded directly into R using the Rsamtools package. Distinct read sequences and the number of times they occurred in the data were stored for each sample. Reads containing indels were filtered out using the read cigar string obtained from the alignment. The number of SNPs per read between individual reads and the template sequence was calculated using the stringdist package. Reads that occurred in the WT samples and were detected at least five times were filtered out to control for background mutations. For all non-WT samples, we calculated the number of distinct reads for each SNP number, used as a measure of editing diversity. All metrics were depth-normalized by dividing by the number of kiloreads sequenced in each sample in order to correct for bias due to different sequencing depths.

### Sanger Sequencing of Individual Clones and Data Analysis

Canavanine-resistant clones were subjected to the yeast-colony PCR^51^ with the primers can1_wh_fw and can1_wh_rv, and the PCR product was purified and Sanger-sequenced with the primers can1_wh_rv and can1_rv_2. For the adenine deaminase experiments, canavanine-resistant clones were subjected to the yeast-colony PCR with the primers can_500_fw and can_500_rv, and the PCR products were purified and Sanger-sequenced with the same primers (Supporting Information Table S2).

Sanger sequencing reads were aligned using the MAFFT algorithm^61^ as implemented through Benchling. Resulting alignment files in FASTA format were subsequently processed and visualized using the R software package (version 4.1.2) along with the Tidyverse packages^60^ and tidysq.^62^ Nonreference base counts, along with counts for each base substitution, were tallied up for each position on the *CAN1* gene across samples.

## Supporting Information Available

NGS results on the three fragments of the *CAN1* gene after mutagenesis using different combinations of gRNAs along with dCas9-AID*Δ, Sanger sequencing data from single Can^R^ colonies that occurred after targeted *in vivo* mutagenesis with dCas9-AID*Δ, and number of unique NGS reads having a mutation per sample, grouped by the number of SNPs per read (PDF)
gRNAs used in the present study, primers and oligos used in the present study, plasmids used in the present study, and strains used in the present study (XLSX)

## Notes

All code used in the analysis is available at https://github.com/angelolimeta/Base-Editors-Yeast.
