# Supplementary material for: Targeted In Vivo Mutagenesis in Yeast Using CRISPR/Cas9 and Hyperactive Cytidine and Adenine Deaminases
Source: ACS Synth Biol. 2023 Jul 24;12(8):2278–89. doi: 10.1021/acssynbio.2c00690 (PMC10443040; doi:10.1021/acssynbio.2c00690)
Supplement: Supplementary file 1 — sb2c00690_si_001.pdf [file sb2c00690_si_001.pdf]

Targeted *in vivo* mutagenesis in yeast using CRISPR/Cas9 and hyperactive cytidine and adenosine deaminases

Christos Skrekas[1], Angelo Limeta[,2], Verena Siewers[1,2], Florian David[1]
[1]Department of Life Sciences, Chalmers University of Technology, Gothenburg, SE-41296, Sweden
[2]Novo Nordisk Foundation Center for Biosustainability, Technical University of Denmark, DK-2800 Kgs. Lyngby, Denmark

Supplementary Figures

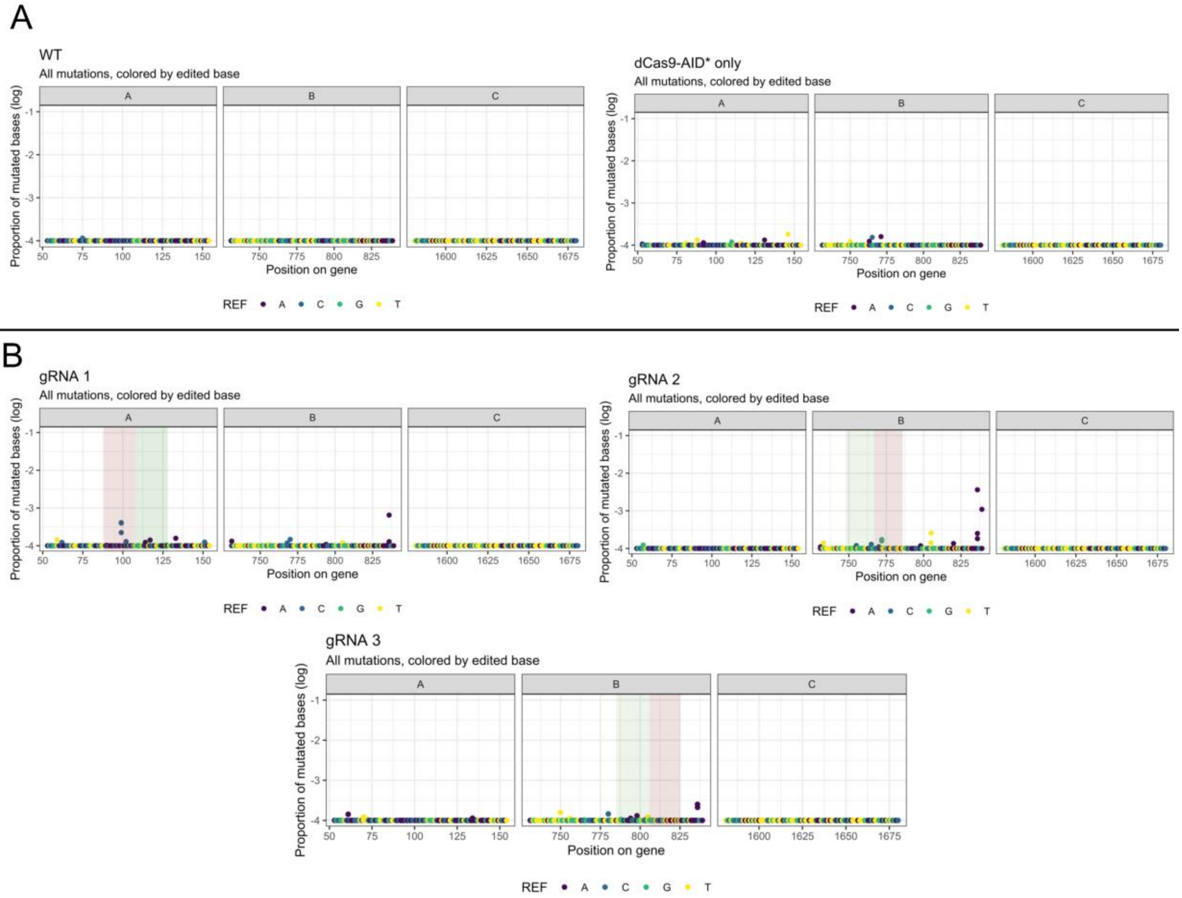

Supplementary Figure 1. NGS results on the three fragments of the *CAN1* gene after mutagenesis using different combinations of gRNAs along with dCas9-AID*Δ. NGS fragments and gRNAs are shown on Figure 1B. The x axis of each graph denotes the gene position and the y axis the proportion of each mutation over the wild type control in logarithmic scale. The -20 bp region from the PAM site of each gRNA is shown in red and the +20 bp region from the PAM site is shown in green. Each reference base that was mutated is shown with a different colour **A.** Mutation spectra of WT samples (left) and samples with only dCas9-AID*Δ (right). **B.** Mutation spectra of the three single gRNAs tested.

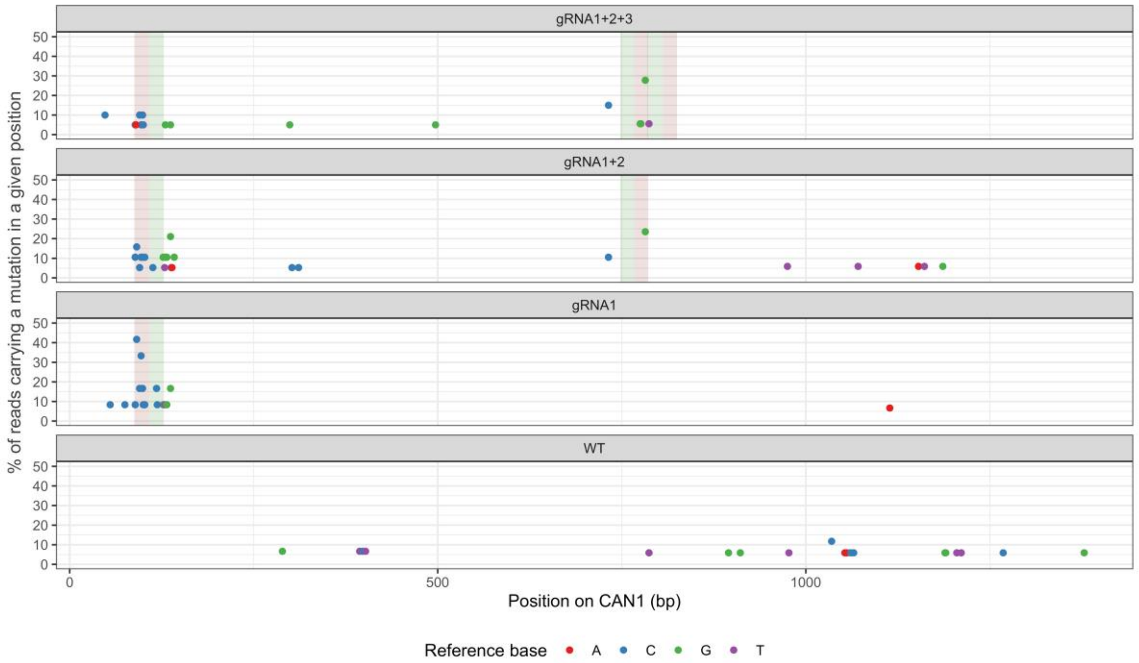

Supplementary Figure 2. Sanger sequencing data from single Can^R colonies that occurred after targeted *in vivo* mutagenesis with dCas9-AID*Δ. 20 single colonies resistant to canavanine from WT experiment, expression of gRNA1 only, multiplexed expression of gRNAs 1 and 2 and multiplexed expression of gRNAs 1, 2 and 3, were selected and the *CAN1* gene sequenced by Sanger sequencing. Mutated bases are plotted with each different reference base denoted with different colours. The x axis denotes the base pair position and the y axis the percentage of reads carrying a mutation at the given position.

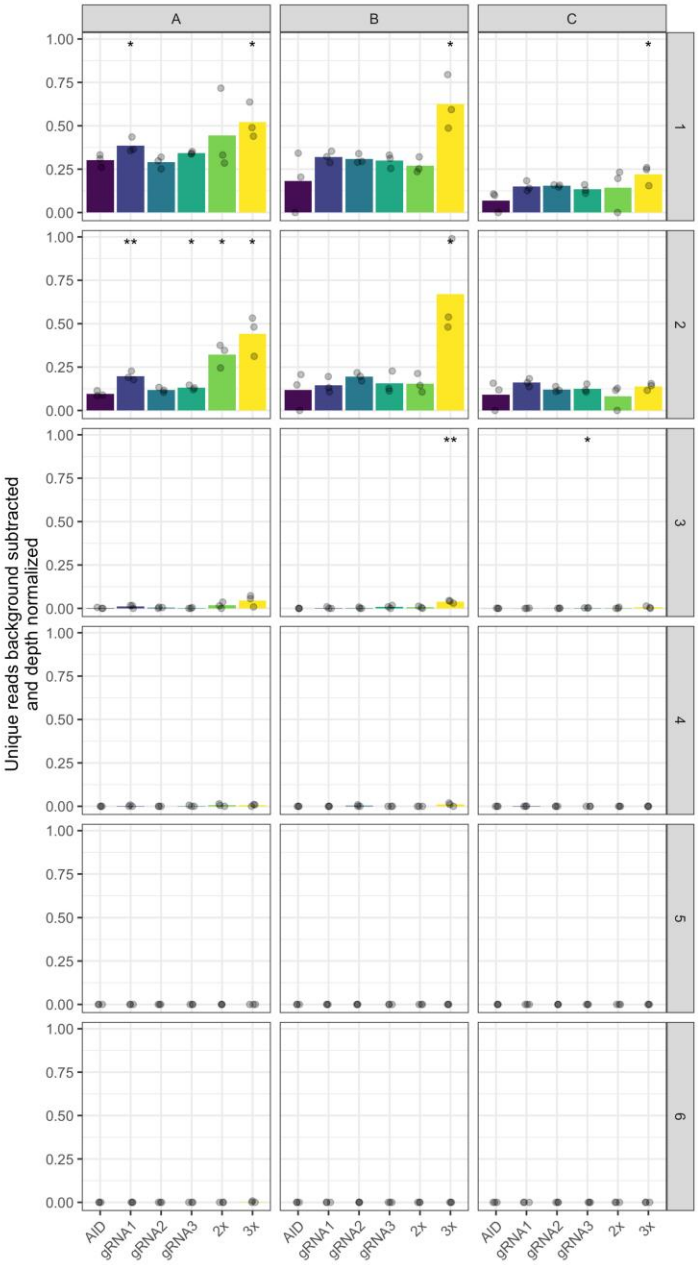

Supplementary Figure 3. Number of unique NGS reads having a mutation per sample, grouped by the number of SNPs per read. The same dataset as in Supplementary Figure 2 was used, but the unique reads are grouped by the total number of SNPs. The AID sample was used as a reference and p-values were calculated for each sample. * p-value < 0.05, ** p-value < 0.01 *** p-value < 0.001.
